# *Chromobacterium violaceum* Isolated from a Wound Sepsis: A Case Study from Nepal

**DOI:** 10.1155/2015/181946

**Published:** 2015-12-16

**Authors:** Shamshul Ansari, Pramod Paudel, Kishor Gautam, Sony Shrestha, Sangita Thapa, Rajendra Gautam

**Affiliations:** ^1^Department of Microbiology, Chitwan Medical College Teaching Hospital, Bharatpur, Chitwan, Nepal; ^2^Department of Medicine, Chitwan Medical College Teaching Hospital, Bharatpur, Chitwan, Nepal

## Abstract

*Chromobacterium violaceum* is a facultative anaerobic, Gram-negative rod, prevalent in tropical and subtropical regions. It enters through the skin injury and is capable of causing severe systemic infections leading to septic shock and multiorgan failure. It has been reported by few authors across the world but this is probably the first case of* Chromobacterium violaceum* isolated from wound sepsis from Nepal. In this study, a pus sample from the infection of a prick injury in the left middle finger was collected from the patient admitted to the intensive care unit. Bacteriological investigations of the pus sample revealed the causative organism to be* Chromobacterium violaceum*. This case study indicates that* Chromobacterium violaceum* can act as a potential cause of wound sepsis that may lead to the septic shock and if not treated timely, the mortality rate can be high as was in this study. Although this organism is very rare, the infection caused requires prompt treatment to minimize the mortality rate. Therefore, we recommend the timely diagnosis and antimicrobial therapy of this infection to combat the consequences led.

## 1. Introduction

*Chromobacterium violaceum* is a Gram-negative, rod-shaped, motile, and facultative anaerobic bacterium that gives positive catalase and oxidase reaction. It grows readily on different bacteriological culture media including nutrient agar (NA), blood agar (BA), chocolate agar (CA), and Muller-Hinton agar (MHA) at 35–37°C [1, 2].* Chromobacterium violaceum* can be isolated from water and soil in tropical and subtropical regions [3]. The organism rarely infects humans; yet, occasionally, the organism can establish a severe systemic infection by entering the bloodstream via an open wound [4]. In most of the cases the organism enters through the breaching of skin, following contamination of soil or water. The effects range from cutaneous lesion to severe sepsis and septic shock progressing rapidly to the death due to the multiorgan failure [5, 6]. We have reported a case of wound sepsis and septic shock caused by* Chromobacterium violaceum* at Chitwan Medical College Teaching Hospital (CMCTH), Bharatpur, Chitwan, Nepal. Probably this is the first reported case of* Chromobacterium violaceum* from Nepal.

## 2. Case Presentation

A 45-year-old male came to the emergency service of Chitwan Medical College Teaching Hospital (CMCTH), a 600-bed referral hospital of central Nepal, at midnight (11:40 pm) with clinical presentation of fever, burning sensation over epigastric region, and difficulty in breathing for two days. There was no nausea and vomiting reported together with normal bowel and bladder. History of patient revealed prick injury in left middle finger by unknown foreign body seven days back. On examination the patient had blood pressure of 100/70 mmHg, pulse rate of 100 beats per minute, and respiratory rate of 24 breaths per minute and the patient was anxious.

The close observation of middle finger enabled prick injury, swollen and tense, extension to middle and proximal phalynx, collection of pus, peeling of skin, sloughing off, presence of sinuses, and discharge of pus material. The patient was provisionally diagnosed as a case of wound sepsis with septic shock together with acute kidney injury (previously called renal failure). The patient underwent pus drainage and pus investigation for bacteriological profile (culture and antibiotic sensitivity result) and surgical consultation.

The patient was admitted to intensive care unit (ICU) and after 2 hours the blood pressure was remarkably decreased and central venous pressure (CVP) line inserted, and inotropic support (noradrenaline) was started. The antimicrobial regime of piperacillin/tazobactam, flucloxacillin, and metronidazole was immediately started together with IV fluid (6 pints of dextrose normal saline over 24 hours), pantoprazole, and hydrocortisone.

The collected pus sample was subjected to bacteriological investigations (Gram's staining, culture, and sensitivity testing). First, the pus sample was inoculated on MacConkey agar (MA), blood agar (BA), and chocolate agar (CA) and then Gram's staining was performed. All the inoculated plates were incubated at 37°C for overnight and, the next day, numerous growths of dark violet colonies with no hemolysis were observed. Subculture on Muller-Hinton agar (MHA) showed notable characteristic feature of nondiffusible, dark violet metallic pigment production.

The growth was further carried out for biochemical testing for catalase, oxidase, triple sugar iron (TSI) agar, sulphide indole motility (SIM), urea hydrolysis, citrate utilization, methyl red, and Voges-Proskauer and the results of biochemical tests were described in Table 1. We felt difficulty in interpreting the result of oxidase test because of the violet color of colonies. However, this difficulty was overcome by incubating the agar plate anaerobically and there was no or minimum pigment formation as pigment production is inhibited by anaerobic environment. After a few hours under aerobic conditions, the colonies turned violet again.

Antibiotic susceptibility testing was performed on dark violet colonies for a range of antimicrobials (amikacin, gentamicin, ceftriaxone, ciprofloxacin, cotrimoxazole, piperacillin-tazobactam, meropenem, and amoxicillin-clavulanic acid) on MHA and the result of susceptibility test was interpreted according to the CLSI guidelines for non-Enterobacteriaceae Gram-negative bacteria [7].

On admission, the hematological investigations showed a decreased total leukocyte count with low hemoglobin (Table 2). The blood chemistry investigations also showed remarkable results except for random blood sugar concentration of 114.0 mg/dL (reference range 70–140 mg/dL) (Table 3). The presence of albumin in urine routine examination revealed the renal dysfunction whereas arterial blood gas disclosed severe metabolic acidosis. The electrocardiogram (ECG) of patient revealed the sinus tachycardia and chest X-ray showed infiltrates in bilateral middle and lower zone.

The patient was subjected to debridement of abscess, the sloughed off tissues and necrotic material were removed, and the wound was washed with Betadine and hydrogen peroxide and then with normal saline. Betadine packing and dressing were applied and advised for daily dressing. Despite these vital procedures, the patient's condition worsened and death was declared after 21 hours of arrival to the hospital because of septic shock with multiple organ failure. The results of bacteriological investigation were yet to come.

The antibiotic sensitivity report revealed that the isolate was susceptible to all subjected antimicrobials. Based on the bacteriological findings the causative agent was identified as* Chromobacterium violaceum*.

## 3. Discussion

Human infections caused by* Chromobacterium violaceum* are uncommon as very few cases have been reported in scientific literatures [2, 5] and we could not find any published literature from Nepal. To the best of our knowledge, this is the first case of* Chromobacterium violaceum* reported from wound sepsis from Nepal. However, few cases have been published from India, Vietnam, Brazil, and Argentina [8–11].

Apart from the several useful compounds (for environmental detoxification, bioprospecting, pest control, and therapeutics) this bacterium can produce violacein, a pigment that displays cytotoxic and antibacterial activity [5, 12]. The violacein production is unique to* Chromobacterium violaceum*, differentiating the organism from other tropical, soil dwelling organisms [13].

The dominant route of infection for this pathogen is through exposure of injured skin to contaminated water or soil, with effects ranging from cutaneous lesions and visceral abscesses to severe sepsis, which progresses rapidly to death [6]. One report from United States [14] and one report from India [2] described fatality rate to be as high as 57% in each study. In our case study, probably the pricking injury and the entry of organism may be while working in the paddy field filled with stagnant water contaminated with this organism. The quick evolution of disease and the antibiotic treatment failure result in a mortality rate of over 60% [10]. In this case study too, the sepsis was reported which terminated in the death of patient after 21 hours of arrival to the hospital due to fast progression of fatal consequences of sepsis and septic shock.

## 4. Conclusions

Our case report reinforces the quick and timely treatment of infections caused by* Chromobacterium violaceum*. Although this organism is very rare, the consequences developed due to the infection of this organism can be highly fatal. Thus, from this case study we recommend rapid diagnosis and prompt treatment of infections caused by* Chromobacterium violaceum*.

## Figures and Tables

**Table 1:** Bacteriological investigations and their results.

Bacteriological parameters	Results
Gram's stain	Plenty of pus cells and presence of Gram-negative rods
Bacterial growth on MA, BA, and CA	Round, smooth, convex, butyrous, medium size, dark violet colonies, nonlactose fermenting, nonhemolytic, notable production of nondiffusible, dark violet metallic pigment (violacein) in MHA
Triple sugar iron (TSI) agar	
Acid production in slant	Negative
Acid production in butt	Positive
Hydrogen sulphide production	Negative
Gas production	Negative
Glucose fermentation	Positive
Lactose and/or sucrose fermentation	Negative
Alkali production in slant	Positive
Sulphide indole motility (SIM)	
Motility	Positive
Hydrogen sulphide production	Negative
Indole production	Negative
Urea hydrolysis test	Negative
Citrate utilization test	Negative
Catalase test	Positive
Oxidase test	Positive
Methyl red test	Negative
Voges-Proskauer test	Negative

**Table 2:** Hematological investigations and their results.

Parameters	Results	Reference range
Total leukocyte count	2,900/*μ*L	4,000–11,000/*μ*L
Differential leukocyte count		
Neutrophils	88%	40–70%
Lymphocytes	10%	20–40%
Monocytes	01%	2–10%
Eosinophils	01%	2–6%
Basophils	00	0-1%
Platelets count	2,15,000/*μ*L	1,50,000–4,00,000/*μ*L
Hemoglobin (Hb)	9.4 gm/dL	12–18 gm/dL

**Table 3:** Blood chemistry investigations and their results.

Parameters	Results	Reference range
Random blood sugar	114.0 mg/dL	70–140 mg/dL
Blood urea	77.0 mg/dL	15–45 mg/dL
Creatinine	1.8 mg/dL	0.4–1.4 mg/dL
Sodium	127.0 mmol/L	135–150 mmol/L
Potassium	3.5 mmol/L	3.5–5.5 mmol/L
SGPT	537.0 IU/L	<42 IU/L
SGOT	830.0 IU/L	<37 IU/L
ALP	224.0 IU/L	64–306 IU/L
Serum amylase	18.0 IU/L	25–115 IU/L
Serum lipase	169.0 IU/L	73–393 IU/L
Troponin-I	Negative	—
